# Patterns of health care utilization preceding a colorectal cancer diagnosis are strong predictors of dying quickly following diagnosis

**DOI:** 10.1186/1472-684X-14-2

**Published:** 2015-01-20

**Authors:** Robin Urquhart, Grace Johnston, Mohamed Abdolell, Geoff A Porter

**Affiliations:** Department of Surgery, Dalhousie University, Halifax, NS Canada; Cancer Outcomes Research Program, Dalhousie University/Capital District Health Authority, Halifax, NS Canada; School of Health Administration, Dalhousie University, Halifax, NS Canada; Department of Diagnostic Radiology, Dalhousie University, Halifax, NS Canada

**Keywords:** Digestive system neoplasms, Palliative care, Diagnosis, Delivery of health care

## Abstract

**Background:**

Understanding the predictors of a quick death following diagnosis may improve timely access to palliative care. The objective of this study was to explore whether factors in the 24 months prior to a colorectal cancer (CRC) diagnosis predict a quick death post-diagnosis.

**Methods:**

Data were from a longitudinal study of all adult persons diagnosed with CRC in Nova Scotia, Canada, from 01Jan2001-31Dec2005. This study included all persons who died of any cause by 31Dec2010, except those who died within 30 days of CRC surgery (n = 1885 decedents). Classification and regression tree models were used to explore predictors of time from diagnosis to death for the following time intervals: 2, 4, 6, 8, 12, and 26 weeks from diagnosis to death. All models were performed with and without stage at diagnosis as a predictor variable. Clinico-demographic and health service utilization data in the 24 months pre-diagnosis were provided via linked administrative databases.

**Results:**

The strongest, most consistent predictors of dying within 2, 4, 6, and 8 weeks of CRC diagnosis were related to health services utilization in the 24 months prior to diagnosis: i.e., number of specialist visits, number of days spent in hospital, and number of family physician visits. Stage at diagnosis was the strongest predictor of dying within 12 and 26 weeks of diagnosis.

**Conclusions:**

Identifying potential predictors of a short timeframe between cancer diagnosis and death may aid in the development of strategies to facilitate timely and appropriate referral to palliative care upon a cancer diagnosis.

## Background

In Canada, colorectal cancer (CRC) has the second and third highest cancer mortality rates for men and women, respectively, with a five-year relative survival of 65% [1]. Stage at diagnosis is the most important prognostic factor for persons diagnosed with CRC, with higher stage at diagnosis associated with decreased probability of survival [2]. In Nova Scotia (NS), ~20% of patients are diagnosed at stage IV, when treatment with curative intent is rarely a viable option [3]. Furthermore, stage-specific five-year relative survival ratios reveal that many persons diagnosed with less advanced disease, wherein the treatment intent is typically cure, will die *of* or *with* CRC.

Palliative care program (PCP) enrolment is a widely accepted quality indicator of end-of-life care [4, 5]. Persons diagnosed with CRC and at risk of dying should benefit from timely enrolment in a PCP. Our research in NS [6–9], as well as that of others’ [10], has shown that persons who die quickly following a cancer diagnosis are less likely to access PCPs. Several authors have highlighted the importance of studying populations with lower rates of PCP enrolment in order to enhance access to these important services [11–13].

Understanding the predictors of a quick death following diagnosis may improve timely access to PCPs. Specifically, identifying factors prior to diagnosis that are predictive of dying quickly after a cancer diagnosis may help with early identification of persons who would benefit from early formal palliative care services and perhaps should be targeted for a PCP consultation upon diagnosis. Patient demographics, co-morbidities, and health care utilization prior to diagnosis may be helpful in this regard as they represent potential indicators or “flags” that can be assessed in an efficient manner and at a population-level due to their availability in large administrative databases [14]. These factors also align with the ‘groups’ of predictors classified by Walshe et al. [13] in relation to access to palliative care services.

The objective of this study was to explore whether factors in the 24 months prior to a CRC diagnosis predict a quick death post-diagnosis using classification and regression tree (CART) analysis. CART analysis has been used in health research to explore health service utilization [8, 15, 16] and to predict health outcomes [17–19]. We previously used CART analysis to identify combinations of factors that, together, identified subpopulations with low rates of PCP enrolment and presented specific areas of potential intervention [8]. In this study, we continue to explore the utility of CART analysis for identifying predictors, and combinations of predictors, that can further our capacity to understand and improve access to palliative services.

## Methods

### Study setting and population

Data for this population-based study are from a large longitudinal, linked administrative database study of all adult persons (≥20 years of age) diagnosed with CRC in NS from 01Jan2001 to 31Dec2005. Personnel from the NS Cancer Registry (NSCR) identified all CRC cases diagnosed in NS in the time period, and undertook a comprehensive chart review to stage this cohort using the Collaborative Stage (CS) Data Collection System [20]. Histological classification was determined by International Classification of Diseases (ICD) for Oncology codes [21]: ICD-O3 C18 (excluding C18.1), C19, and C20. Cases diagnosed based on death certificate or autopsy were excluded. If persons had >1 CRC diagnosis in the time period, only one case per patient was retained (the specific rules for retaining cases are reported elsewhere [22]). This cohort (n = 3510) was anonymously linked at the patient-level to 14 administrative health databases, providing a comprehensive data source that included clinico-demographic and health service utilization data on all persons with a CRC diagnosis in the province. Further details related to this larger study, including descriptions of the various linked databases and the data linkage process, are reported elsewhere [22]. Ethical approval was obtained from the Capital District Health Authority’s Research Ethics Board.

For the present study, we included all persons in the larger cohort who died of any cause by 31Dec2010. We excluded those who died within 30 days of CRC surgery to remove unexpected deaths arising due to post-operative complications. This resulted in a study cohort of 1885 decedents.

### Variables

Multiple linked databases provided patient and health service utilization data for the 24 months (2 years) prior to CRC diagnosis. The dependent variable was the length of time from CRC diagnosis to death. This variable was analysed as a dichotomous variable (death within various discrete time intervals [yes/no]) and as a continuous variable (in days). Diagnosis and death data were obtained from the NSCR.

The predictor (or independent) variables were all from the 24 months prior to CRC diagnosis, plus stage at diagnosis. Demographic variables included: sex, age at diagnosis (continuous variable, in years), health region, location of residence (urban/rural, using the metropolitan influence zone classification [23]), material and social deprivation indices [24], frailty (yes/no), organ failure (yes/no), and residence in a long-term care facility (yes/no). The presence of frailty and organ failure was computed using algorithms created by Fassbender and colleagues [25], who used a literature review, expert opinion, and cluster analyses to define various causes of death categories (sudden death, terminal illness, organ failure, frailty, and other). The categories ‘organ failure’ and ‘frailty’ are comprised of groups of specific comorbid conditions. Examples of illnesses included in frailty were dementia, Alzheimer’s disease, and Parkinson’s disease; examples of illnesses included in organ failure were chronic obstructive pulmonary disease and congestive heart failure. A person was considered a long-term care facility resident if he/she had at least one physician visit within the last six months of life at a long-term care facility.

Additional socio-demographic variables, obtained from the 2001 census data and linked to patients’ enumeration areas (the smallest geographic unit for which census data are available) were: median household income, unemployment (proportion unemployed), education (proportion without a high school diploma), living arrangement (proportion living alone), marital status (proportion separated, divorced, widowed), single parent (proportion of single parent families), elderly (proportion age 80+; proportion age 85+), immigrants (proportion who are immigrants; proportion who are recent immigrants; proportion who are first generation immigrants), Aboriginal (proportion who are Aboriginal), Black (proportion who are Black), and Francophone (proportion who are Francophone). All census-derived variables were analysed with the proportion represented as a continuous variable.

Health service utilization variables were: number of hospital admissions, total number of days in hospital, number of Emergency Room (ER) visits, number of family physician (FP) visits, number of specialist visits, number of physician visits in a long-term care facility (any physician type), continuity of FP care, and continuity of specialist care. All health service utilization variables were analysed as continuous variables. Cancer site (colon, rectal) was also included as a predictor variable.

Stage at diagnosis was categorized as CS-derived TNM stage I, II, III, IV, or unknown. All variables were extracted from, or computed using variables extracted from, the following administrative databases: NSCR, physicians’ billings, hospital discharge abstracts, and national census data.

### Statistical analysis

Binary recursive partitioning is implemented using the CART algorithm. When the outcome is categorical, classification trees are generated that predict the class of a categorical outcome measure (e.g., death within a certain timeframe). When the outcome is continuous, regression trees are generated that predict the value of the continuous outcome measure (e.g., length of time from CRC diagnosis to death). Tree models are generated by repeated binary partitioning of a population, based on a set of predictor variables, such that the cases in the descendant subsets are increasingly more similar within those subsets, so that the overall tree deviance becomes smaller with each successive split. Splitting stops when the cases in a subset are either entirely, or almost entirely, of the same class or the same value, or when further splitting does not improve discrimination between cases (i.e. does not substantially reduce deviance). When a descendant subset of cases cannot be split any further, that subset is labeled as a ‘leaf’ in the tree. The final set of leaves comprises disjoint sets of cases, with each set being characterized by a series of binary decision rules based on a set of predictor variables [26, 27]. As part of this methodology, the algorithm considers all possible splits related to the data, but only selects the optimal splits. Each split is determined in a data driven way, with lower splits all conditional on the prior splits. Researchers do not select or identify *a priori* split-points for each predictor variable; rather, the algorithm selects the split-point that is optimal using a goodness of fit criterion. Strengths of CART methodology include its ability to process large numbers of predictor variables simultaneously, even with considerable interaction amongst variables [28], and the final model is simply interpreted as a set of binary decision rules that together comprise a decision tree and can be implemented intuitively and simply in the clinical setting.

CART models were used to examine pre-CRC diagnosis predictors of time from diagnosis to death using the following discrete time intervals: 2, 4, 6, 8, 12, and 26 weeks from diagnosis to death. Various time intervals were used since there is no agreement in the literature on what constitutes a “quick” death following a cancer diagnosis. All models were run with and without stage at diagnosis as a predictor variable. These models optimally selected split-points identified from across the set of predictor variables, thereby partitioning the data into increasingly more homogeneous, mutually exclusive subsets. All analyses were performed using the R system for statistical computing [29].

## Results

Of this decedent population, 865 (45.9%) were female; 1150 (61.0%) were urban residents; 192 (10.2%) were residents of a long-term care facility; and 183 (9.7%), 435 (23.1%), 467 (24.8%), 668 (35.4%), and 132 (7.0%) were diagnosed with stage I, II, III, IV, and unknown cancer, respectively. The median (range) age at diagnosis was 75.0 (21–101) years. Table 1 depicts the number and percentage of decedents who died within each time interval examined.

**Table 1:** **Number and percentage of decedents who died within each time interval**

Time interval	n	%
2 weeks	103	5.5
4 weeks	188	10.0
6 weeks	256	13.6
8 weeks	308	16.3
12 weeks	402	21.3
26 weeks	578	30.7

Stage at diagnosis was not a strong predictor of dying within 2, 4, 6, and 8 weeks of diagnosis. Stage at diagnosis was the strongest predictor of dying within 12 and 26 weeks (3 and 6 months, respectively) of CRC diagnosis. The strongest, most consistent predictors of dying within 2, 4, 6, and 8 weeks of CRC diagnosis were related to health services utilization in the two years prior to diagnosis: i.e., number of specialist visits, total number of days spent in hospital, and number of FP visits. For all four of these time intervals, having <8 specialist visits in the two years prior to diagnosis was the strongest predictor of dying within the time period. In addition, frailty was a strong predictor of dying within 4 and 6 weeks of CRC diagnosis.

**Figure 1:**
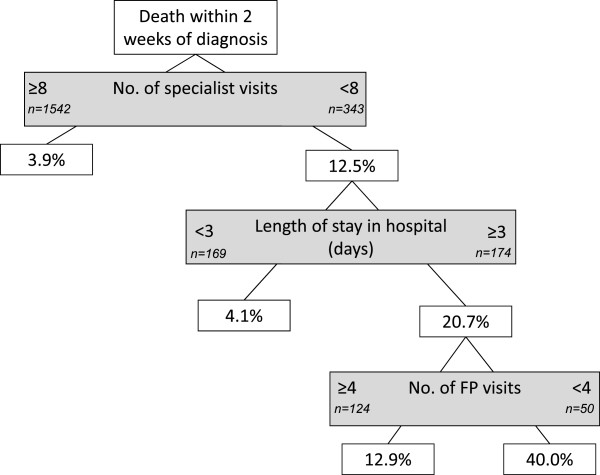
**CART tree for death within 2 weeks of diagnosis.** 103 (5.5%) patients were diagnosed with CRC within 2 weeks of their death. % indicates proportion with those characteristics who died.

**Figure 2:**
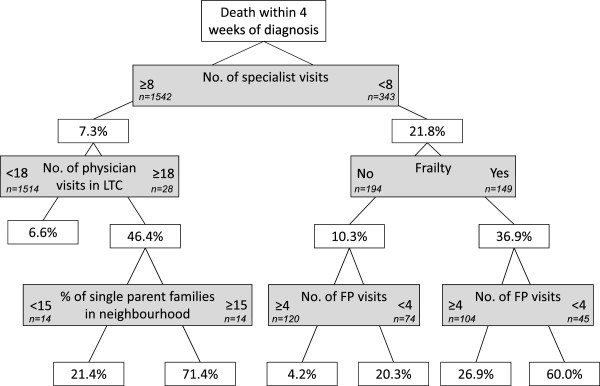
**CART tree for death within 4 weeks of diagnosis.** 188 (10.0%) patients were diagnosed with CRC within 4 weeks of their death. % indicates proportion with those characteristics who died. [LTC = long-term care facility].

**Figure 3:**
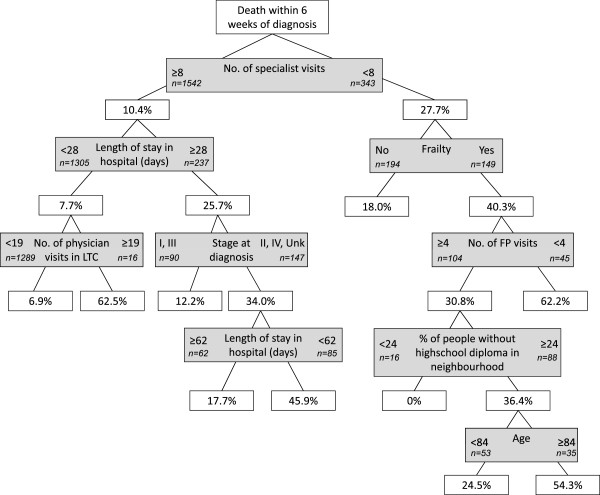
**CART tree for death within 6 weeks of diagnosis.** 256 (13.6%) patients were diagnosed with CRC within 6 weeks of their death. % indicates proportion with those characteristics who died. [LTC = long-term care facility].

**Figure 4:**
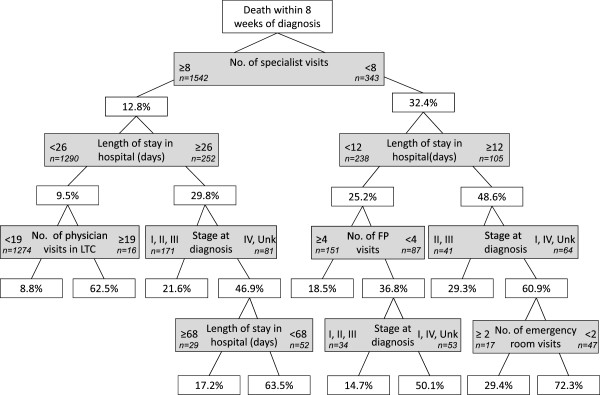
**CART tree for death within 8 weeks of diagnosis.** 308 (16.3%) were diagnosed with CRC within 8 weeks of their death. % indicates proportion with those characteristics who died. [LTC = long-term care facility].

**Figure 5:**
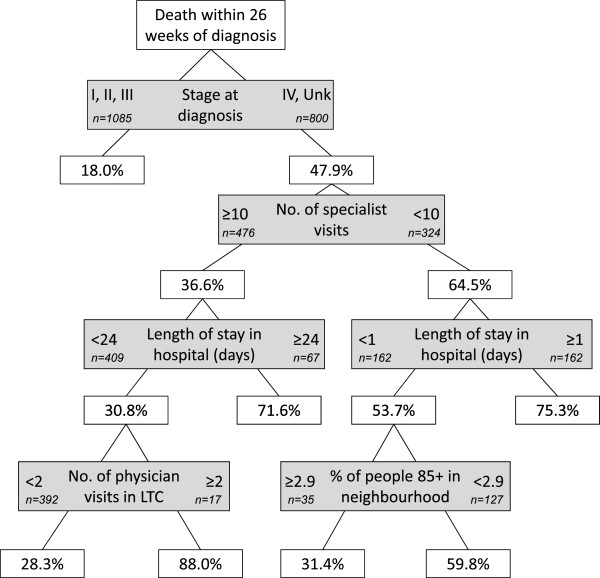
**CART tree for death within 26 weeks (6 months) of diagnosis.** 578 (30.7%) were diagnosed with CRC within 26 weeks of their death. % indicates proportion with those characteristics who died. [LTC = long-term care facility].

Figures 1, 2, 3, 4 and 5 present the CART trees for the time intervals 2, 4, 6, 8, and 26 (6 months) weeks, respectively. All models presented included stage at diagnosis as a predictor variable, though this factor was *not* a predictor of dying within 2 and 4 weeks of diagnosis (and only a moderate predictor of dying within 6 and 8 weeks of diagnosis). Figure 1 shows that among those decedents who had <8 specialist visits, spent ≥3 days in hospital, and had <4 family physician visits in the 2 years prior to diagnosis, 40.0% died within 2 weeks of diagnosis. Figure 2 shows that among those who had <8 specialist visits, were frail, and had <4 family physician visits in the 2 years prior to diagnosis, 60.0% died within 4 weeks of diagnosis. From Figure 5, it can be seen that among those diagnosed with stage IV or unknown stage with <10 specialist visits and ≥1 day in hospital in the 2 years prior to diagnosis, 75.3% died within 26 weeks (6 months) of diagnosis.

The CART trees also revealed sub-populations with low rates of dying quickly post-CRC diagnosis. Figure 1 reveals that study decedents with high rates of specialist visits and little time spent in hospital preceding diagnosis had low rates of dying within 2 weeks of a CRC diagnosis. From Figure 2, decedents with the lowest rate (4.2%) of dying within 4 weeks of CRC diagnosis were those who had few specialist visits (<8), but were not frail and had more family physician visits (≥4) in the 2 years preceding diagnosis.

## Discussion

This study used CART methods to explore whether factors in the 24 months prior to a CRC diagnosis predict a quick death post-diagnosis. Identifying pre-diagnosis factors that are predictive of dying within a short time interval following a cancer diagnosis should help us understand whether certain individuals (or groups of individuals) should be targeted for consideration of prompt PCP referral upon diagnosis. We found that fewer physician visits and greater time spent in hospital were strong predictors of dying quickly (i.e., within 8 weeks) after CRC diagnosis whereas stage at diagnosis was not a strong predictor of dying quickly. These findings represent a novel contribution to the palliative care literature; we are unaware of any prior study that has investigated pre-diagnosis predictors of dying quickly following a cancer diagnosis.

The strongest and most consistent predictors of dying within a short timeframe following CRC diagnosis were related to health service utilization in the two years prior to diagnosis. Specifically, fewer visits to specialists and FPs, and longer cumulative time spent in hospital, prior to diagnosis were relatively consistent in terms of identifying sub-populations at risk of dying quickly following CRC diagnosis. The optimum split-points for the factors sometimes differed depending on the time interval studied. Together, these factors may be indicative of poor disease management, with lower use of physician services and higher use of hospital services when conditions or illnesses are not adequately diagnosed and managed out of hospital, leading to deterioration in health and requiring hospital admission. Alternatively, the burden of illness or severity of symptoms experienced by these decedents might have made community-based disease management not feasible, or simply quite difficult. In the latter case, accessing outpatient clinics may have been problematic for these individuals due to high disease burden, resulting in them eventually being admitted to hospital when they could no longer stay at home (or at a long-term care facility). While the exact reasons for this pattern of health care use are unknown, identifying such predictive patterns of health services may provide an opportunity to develop and target interventions to improve patient care [14]. The limited contact with healthcare providers in the sub-populations who die relatively quickly following CRC diagnosis highlights the importance of ‘upstream’ care in identifying and managing health issues that might increase an individual’s vulnerability for adverse outcomes.

One health issue may be frailty. In this study, frailty was a predictor of dying within 4 and 6 weeks of CRC diagnosis. Numerous studies have shown that frailty is an independent risk factor for major morbidity and mortality in various patient populations [30–33]. Notwithstanding the conceptual, definitional, and measurement issues associated with assessing frailty [34], most researchers agree that frailty increases a person’s vulnerability to adverse outcomes [35], even in the presence of relatively minor stressors [36]. Although frailty is likely highly correlated with age [35], in this study, age was not a strong predictor (nor a weak consistent predictor) of dying quickly after CRC diagnosis. However, a greater number of physician visits in a long-term care facility was a predictor of dying within 4 weeks of CRC diagnosis (Figure 2). It is possible that the CRC diagnosis was an incidental or unanticipated diagnosis for persons with a complex set of symptoms or multi-morbidity.

As demonstrated in the CART trees, our analyses also defined subpopulations, based on interactions amongst predictor variables, with *low* rates of dying quickly after a CRC diagnosis. The decedents with the lowest rate of dying within four weeks of diagnosis were those with few specialist visits, but were not frail and had four or more family physician visits in the two years before diagnosis (Figure 2). This is interesting given that fewer specialist visits (<8) was often the strongest predictor of dying within a short timeframe. While we can only speculate on possible explanations for this finding, one is that the interaction of these particular factors is indicative of well health. That is, decedents in this subgroup may have been relatively healthy prior to their CRC diagnosis with few comorbid conditions or complex medical needs, at least not severe enough to require high specialist care, and were receiving regular primary care (wherein any comorbid conditions may have been well-managed). Thus, their CRC diagnoses and management may not have been complicated by other health/illness factors.

A number of socio-demographic characteristics that are frequently associated with inequities in cancer care and outcomes, such as income, education, sex, and geographic location, were not key predictors of time from cancer diagnosis to death in this study. In a review of Canadian literature on inequities in cancer care [37], income, geography, sex, and age were frequently associated with both access to palliative care services as well as mortality outcomes. For instance, when logistic regression methods are used, persons diagnosed with cancer from rural areas have been shown to have poorer access to palliative care services [7], while persons with lower socioeconomic status have been shown to have an increased likelihood of dying within six months of diagnosis [38]. We employed a different analytic methodology to identify and group potential predictors of an outcome, which may account for the differences with prior studies.

Certainly, early and complete assessment of a person’s health is important in ensuring timely palliative support. Even those who die quickly following a cancer diagnosis should benefit from care that focuses on pain and symptom management, psychosocial well-being, and bereavement support, which are integral features of palliative care. However, care providers are challenged to consistently and accurately anticipate death at the time of diagnosis [39–41], thus potentially compromising timely access to PCPs. This may be especially pertinent if the condition(s) leading to a quick death are outside the scope of a care provider’s own medical specialty. That stage at diagnosis was not a strong predictor of a quick death following diagnosis highlights the complexity of anticipating impending death. This study represents an important step toward identifying and elucidating potential predictors of a short timeframe between a cancer diagnosis and death that can aid in the development of flags or algorithms that ultimately facilitate care providers’ decision-making with respect to palliative care referral. The utility of pre-diagnosis factors, especially patterns of health care utilization, for predicting a quick death following a cancer diagnosis requires further study.

This study has a number of strengths. First, we examined all persons who were diagnosed with and died of or with CRC in one province. Thus, we identified predictors of a quick death at a population level (versus in a clinical subset of patients). Second, unlike regression analyses, CART can handle large numbers of predictor variables [42] and identifies optimal split-points, eliminating the need to categorize variables *a priori*. As we demonstrated in a prior study [8], by partitioning the data through recursive splitting, CART analysis defines target subpopulations for further investigation and intervention in a more straightforward manner than multiple logistic regression. That is, CART methodology allows one to identify subpopulations that are defined by interactions amongst predictor variables (as shown in the figures). In addition, the final CART model is presented as a decision tree that lays out a set of intuitive decision rules easily used in the field. There are also limitations. The predictor variables were limited to data available in large administrative databases; thus, clinical factors (e.g., functional status, severity of co-morbid illness) were not included in the analyses. Nonetheless, the identification of predictors from administrative databases allows assessment on a population level and provides a means for developing a monitoring system that facilitates the early identification of persons who may benefit from timely referral to palliative care services.

## Conclusion

The strongest and most consistent predictors of dying quickly following a CRC diagnosis were related to health service utilization in 24 months prior to diagnosis whereas stage at diagnosis became the strongest predictor of dying within 12 and 26 weeks after a CRC diagnosis. The pattern of health care utilization predictive of dying quickly following diagnosis may highlight a situation of poor disease management, frailty, or multi-morbidity. Further research is required to corroborate these findings and assist in the development of algorithms that may facilitate timely and appropriate referral to PCPs upon a cancer diagnosis.
